# Impact of Treatment Package Time on Survival in Patients with Head and Neck Adenoid Cystic Carcinoma

**DOI:** 10.3390/cancers18050816

**Published:** 2026-03-03

**Authors:** Emile Gogineni, Ela Kini, Demond Handley, Yevgeniya Gokun, Sung Jun Ma, David J. Konieczkowski, Darrion L. Mitchell, Simeng Zhu, John C. Grecula, Sachin R. Jhawar, Marcelo Bonomi, Priyanka Bhateja, Kyle K. VanKoevering, Ricardo L. Carrau, James W. Rocco, Arnab Chakravarti, Dukagjin M. Blakaj, Matthew Old, Sujith Baliga, Rafi Kabarriti

**Affiliations:** 1 Department of Radiation Oncology, The Ohio State University Wexner Medical Center, Columbus, OH 43210, USAdarrion.mitchell@osumc.edu (D.L.M.);; 2 Hunter College High School, New York, NY 10128, USA; 3 Center for Biostatistics, The Ohio State University Wexner Medical Center, Columbus, OH 43210, USA; 4 Department of Medical Oncology, The Ohio State University Wexner Medical Center, Columbus, OH 43210, USA; 5 Department of Otolaryngology, The Ohio State University Wexner Medical Center, Columbus, OH 43210, USA; 6 Department of Radiation Oncology, Montefiore Medical Center, Albert Einstein College of Medicine, Bronx, NY 10461, USA

**Keywords:** head and neck cancer, radiation therapy, radiotherapy, surgery, adenoid cystic carcinoma, salivary gland, malignancy, malignant, treatment package time, duration, delay, delays, time, treatment initiation

## Abstract

Background/Objectives: Treatment delays have been shown to be associated with overall survival (OS) in head and neck squamous cell carcinomas (HNSCCs). Given the slow tumor growth kinetics of adenoid cystic carcinoma (ACC), it is unclear if delays have a similar impact in this tumor histology. Methods: We queried the National Cancer Database for patients diagnosed with non-metastatic ACC between the years 2004 and 2019 and treated with surgery followed by RT. A multivariable Cox regression model was used to examine the associations between the time from diagnosis to surgery, the duration of RT, and OS. Results: A total of 1449 patients were included for analysis. Increased time from diagnosis to surgery (HR: 1.02, 95% CI: 1.01–1.03, *p* < 0.001) and duration of RT (HR: 1.14, 95% CI: 1.04–1.25, *p* = 0.004) were associated with worse survival on UVA, while time from surgery to RT start was not (*p* = 0.647). Increased duration of RT (aHR: 1.13, 95% CI: 1.03–1.24, *p* = 0.012) remained significantly associated with OS on multivariable analysis, while time from diagnosis to surgery (aHR: 1.00, 95% CI: 0.98–1.02, *p* = 0.979) did not. Conclusions: Delays in treatment initiation and in the interval from surgery to radiation did not result in clinically significant differences in survival in this analysis, while prolonged duration of radiation therapy was significantly associated with worse survival. These findings are hypothesis-generating and suggest that treatment delays for ACC may have different effects on oncologic outcomes than those for HNSCC; however, prospective data is paramount to verify these results before strong conclusions can be made.

## Simple Summary

Delays in treatment initiation and completion are associated with poor outcomes for head and neck squamous cell carcinomas. Adenoid cystic carcinoma (ACC) is a rare malignancy characterized by slow growth patterns, and thus it is unclear if delays have similar impacts on survival for this type of cancer. This study analyzed outcomes for 1449 patients with non-metastatic ACC treated with surgery and radiation between 2004 and 2019 in the National Cancer Database. After adjustment for relevant clinical and sociodemographic factors, time from diagnosis to surgery and time from surgery to the start of radiation were not associated with clinically meaningful differences in overall survival. In contrast, longer radiation duration was independently associated with worse survival. These results support prioritizing efficient, uninterrupted radiation schedules and addressing barriers that extend the course. Prospective studies are needed to confirm these findings and optimize treatment timing strategies for ACC patients.

## 1. Introduction

The estimated incidence of cancers of the oral cavity, pharynx, and larynx in the United States in 2024 was 71,100, representing 3.6% of all malignancies [1]. The large majority of these cancers in the head and neck are squamous cell carcinomas (HNSCC), while salivary gland tumors represent roughly 6% of head and neck cancers, with an annual incidence in the United States of 2500 cases [2,3]. Salivary gland tumors are classified according to histology using the World Health Organization (WHO) 2005 system [4]. There are over 40 histologies of salivary gland tumors, broadly categorized into benign and malignant subgroups, with each characterized by a unique natural history, prognosis, and pattern of failure [3].

Adenoid cystic carcinoma (ACC) is a rare and aggressive salivary malignancy, accounting for 10% of all salivary gland tumors and roughly 20% of malignant salivary gland neoplasms [5,6]. It has a propensity for perineural and local tumor invasion, as well as late disease recurrence [7,8,9,10,11].

Surgery followed by postoperative radiation therapy (RT) is a commonly employed treatment regimen for both HNSCC and malignant salivary gland tumors and represents the standard-of-care treatment for all cases of non-metastatic ACC [12]. Recent advances in diagnostic technology are transforming the landscape of head and neck cancer detection and management. Hyperspectral imaging (HSI) combined with computer-aided diagnostic (CAD) methods utilizing machine learning algorithms have shown promise for improved tumor detection and margin assessment, with reported accuracies exceeding 90% in head and neck malignancies [13]. While these technological innovations enhance our ability to detect and characterize tumors, fundamental questions regarding optimal treatment timing for specific histologic subtypes remain inadequately addressed in the literature, particularly rare entities like ACC.

Several factors inherent to cancers of the head and neck serve as potential sources of treatment delays, such as the multimodal nature of treatment, need for dental clearance before initiation of RT, and the social determinants of health and comorbidities associated with patients who develop head and neck cancers. These delays can include time from diagnosis to treatment initiation and total treatment package time (TPT), which is defined as the time from surgery to completion of postoperative RT when treating with these two modalities. TPT can be further subdivided into time from surgery to RT initiation and duration of RT from start to completion.

HNSCC has the potential to grow rapidly, increasing the risk of accelerated tumor clonogen repopulation between surgery and RT initiation and during RT [14,15]. Thus, outcomes for HNSCC have a strong association with duration of treatment. Several studies have shown that delays in initiation of treatment and delays between surgery and RT negatively impact rates of control and survival for HNSCC [16,17,18,19,20,21,22,23,24,25,26,27,28].

This association between treatment delays and outcomes has also been shown for other high-risk cancers of the head and neck, such as malignant salivary gland tumors [29]. The American Society of Clinical Oncology (ASCO) guidelines for management of salivary gland malignancies recommend that postoperative RT should begin within 8 weeks of surgery [12]. According to the authors, this recommendation was classified as an informal consensus with insufficient quality of evidence, leading to a moderate strength of recommendation.

The natural history of each subtype of salivary gland tumor varies widely, suggesting that delays may be more impactful for some histologies than others. Despite ACC’s malignant features and high recurrence rates, it is characterized by indolent growth kinetics and a prolonged clinical course, contrasting sharply with the rapid proliferation observed in HNSCC [30]. Accordingly, recurrences typically occur years after initial treatment, with reports of failure 20–30 years after primary diagnosis [31,32].

Given ACC’s fundamentally different growth kinetics, treatment timing principles derived from rapidly proliferating HNSCC may not be directly applicable to ACC. This raises the question of whether delays in the treatment of ACC have similar impacts on survival as they do for HNSCC. To our knowledge, there have been no studies published to date assessing the association between treatment delays and outcomes for ACC, representing a significant knowledge gap in the management of this rare malignancy. Our objective was to analyze the impact of prolonged time to treatment initiation (TTI), time from surgery to RT start, and duration of RT on overall survival (OS) in patients with ACC. We hypothesized that delays in treatment initiation and completion would not have a significant impact on survival for patients with ACC.

## 2. Materials and Methods

### 2.1. Data Source

The National Cancer Database (NCDB) is a joint project of the American College of Surgeons’ Commission on Cancer (CoC) and the American Cancer Society (ACS). It is a hospital-based registry that includes patients from over 1500 CoC-accredited institutions and is estimated to capture roughly 70% of malignancies diagnosed within the United States.

The NCDB was queried for all patients diagnosed with non-metastatic ACC between the years 2004 and 2019 who underwent surgery followed by postoperative RT to a dose of 40–80 Gy, with or without chemotherapy. Patients who did not undergo surgery and/or radiation, whose TPT exceeded 200 days, or whose duration of RT was less than 40 days were excluded from analyses. The 200-day TPT threshold was selected to exclude outlier cases that likely represent unique clinical scenarios (e.g., prolonged recovery from surgical complications, treatment at multiple non-coordinated institutions, or patient-driven delays) that would not reflect typical treatment delivery patterns. This cutoff represents approximately 28 weeks, which is substantially beyond a reasonable timeline when incorporating the 8-week ASCO recommendation for time between surgery and RT start with a standard fractionation scheme of 6–7 weeks of postoperative radiation, allowing for real-world delays while excluding extreme outliers. The 40-day RT duration minimum was implemented to exclude palliative and hypofractionated radiation regimens, ensuring our cohort reflects patients receiving definitive treatment via surgery followed by conventionally fractionated postoperative RT as per standard ACC management. These thresholds are consistent with prior NCDB-based and retrospective studies examining treatment timing in HNSCC [21,33]. Given that all data included was deidentified and obtained from a preexisting, publicly accessible database, the study was exempt from review by our Institutional Review Board.

### 2.2. Study Measures

TTI was defined as the number of days from date of initial diagnosis of ACC, as proven by biopsy, to date of surgery. Time from surgery to RT initiation was defined as the number of days between surgery and the first fraction of RT. RT duration was defined as the number of days from first fraction of RT to the final fraction.

### 2.3. Sociodemographic Characteristics

Sociodemographic data included age, sex, race, ethnicity, insurance, rurality, and zip code-level median household income. In this analysis, race and ethnicity were treated as sociopolitical designations rather than proxies for genetic ancestry [34].

### 2.4. Clinical and Treatment Characteristics

Disease characteristics included the Charlson–Deyo comorbidity score (0, 1, 2, ≥3), primary site of diagnosis, tumor grade (1 or 2, 3 or 4), pathologic T-stage (pT1, pT2, pT3, pT4), and pathologic N-stage (pN0, pN+, no lymph nodes examined). Treatment characteristics included margins of the primary site (negative, positive), radiation dose (Gy), and chemotherapy (yes, no).

### 2.5. Outcome

The primary endpoint was OS. Survival time was calculated from the date of histologic diagnosis of ACC to the earliest of death, last known contact, or the study end date. Individuals who had not died by the end of the observation period were considered censored at their last recorded follow-up.

### 2.6. Statistical Analysis

We described sociodemographic, clinical, and treatment characteristics using standard summary statistics: continuous variables are presented as medians with interquartile ranges, and categorical variables as counts with corresponding percentages. The proportional hazards assumption for each study measure was evaluated using two complementary approaches. First, a Cox proportional hazards model including an interaction term between follow-up time and the study measure was fitted; a statistically significant interaction term indicated potential violation of the proportional hazards assumption. Second, the assumption was assessed using the supremum test for proportional hazards [35].

Univariable Cox regression models were fitted to assess crude associations between the outcome OS and each study measure (TTI, time from surgery to RT, duration of RT), patient sociodemographic, clinical, and treatment characteristics. A multivariable Cox regression model was fitted to assess associations between the outcome OS and each study measure, adjusting for sociodemographic, clinical, and treatment characteristics that were statistically significant in univariable analyses. Because both the time from surgery to RT and duration of RT were dependent on TTI and violated the proportional hazards assumption, both measures were included in models as time-dependent covariates using the counting process [36]. Estimates were adjusted for by age, sex, type of health insurance, rurality, Charlson–Deyo score, margins of the primary site, and chemotherapy. Dose–response relationships between duration of RT and OS were evaluated by fitting a multivariable Cox regression model using a three-knot restricted cubic spline, where estimates were adjusted for by age, sex, form of health insurance, rurality, Charlson–Deyo score, pathologic T-stage, pathologic N-stage, margins of the primary site, radiation dose, TTI, and chemotherapy. The three knots were placed at the 10th, 50th, and 90th percentiles of RT duration. In supplementary analyses, a logistic regression model was fitted to assess the association between receipt of chemotherapy versus no chemotherapy and T-stage, pathologic N-stage, margins of the primary site, and duration of RT. In subgroup analyses, among patients stratified by T-stage, N-stage, and margins of the primary site, univariable Cox regression models were fitted to assess the association between receipt of chemotherapy and OS. For Cox regression models, all estimates are reported as hazard ratios (HRs) along with 95% confidence intervals (95% CI). For logistic regression models, all estimates are reported as odds ratios (ORs) along with 95% CI. All analyses were conducted using SAS 9.4 (SAS Institute, Cary, NC, USA), and a *p*-value of less than 0.05 was the threshold for determining statistical significance.

## 3. Results

### 3.1. Study Population Characteristics

**Figure 1:**
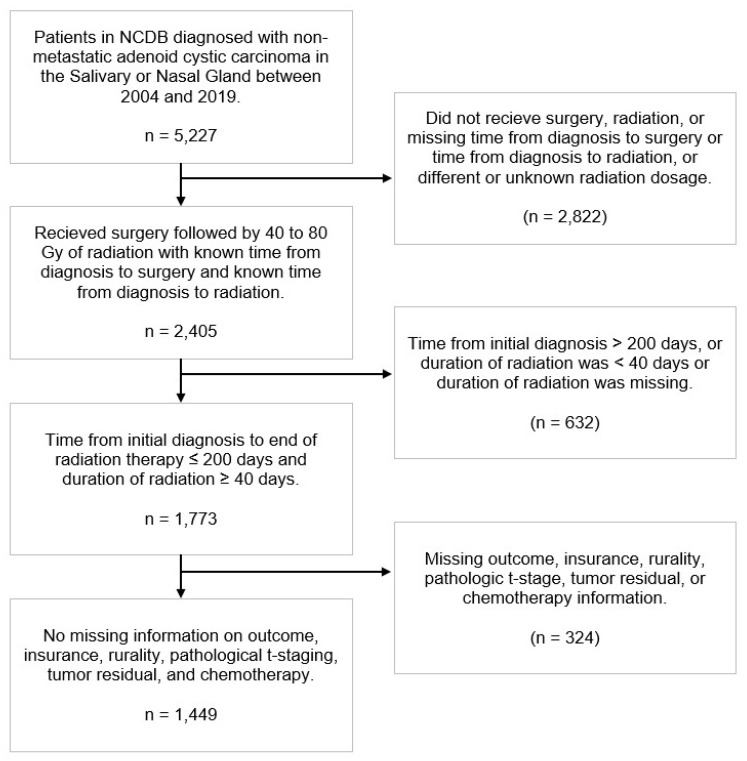
The National Cancer Database (NCDB) included 5227 patients diagnosed with non-metastatic adenoid cystic carcinoma between 2004 and 2019. A total of 1449 patients were included for analysis after applying exclusion criteria.

We identified 5227 patients diagnosed with non-metastatic ACC between 2004 and 2019. As shown in Figure 1, 1449 patients were included for analysis after applying the exclusion criteria. Table 1 summarizes patient demographic, clinical, and treatment characteristics. The median (IQR) age was 58 (47–67), and 1211 (83.6%) patients had a Charlson–Deyo score of 0. A total of 1256 (86.7%) patients lived in metropolitan regions. A total of 845 (58.3%) patients had private insurance, while 434 (30.0%) had Medicare coverage. The most common primary sites included parotid (48.8%), submandibular gland (28.0%), maxillary sinus (9.5%), and nasal cavity (7.2%). The pathologic T-stage distribution was relatively uniform, with 24.2–26.3% of patients classified as pT1, pT2, pT3, and pT4. A total of 197 (13.6%) patients had node-positive disease. A total of 771 (53.2%) patients had positive margins, while 163 (11.2%) received chemotherapy. The median (IQR) radiation dose was 66 (60–66) Gy. The median (IQR) follow-up was 66.7 (38.8–105.4) months.

### 3.2. Univariable Analysis

Univariable analysis (UVA) for OS is provided in Table 2. On UVA, increased TTI (HR: 1.02, 95% CI: 1.01–1.03, *p* < 0.001) and duration of RT (HR: 1.14, 95% CI: 1.04–1.25, *p* = 0.004) were associated with worse survival, while time from surgery to RT start was not significantly associated with OS (HR: 1.01, 95% CI: 0.97–1.04, *p* = 0.647).

Additionally, increasing age (HR: 1.04, 95% CI: 1.03–1.05, *p* < 0.001), urbanity (HR: 1.54, 95% CI: 1.15–2.03, *p* = 0.003), Medicare insurance (HR: 2.48, 95% CI: 2.00–3.08, *p* < 0.001), income < $46,227 (HR: 1.46, 95% CI: 1.06–1.99, *p* = 0.016), Charlson–Deyo score of 1 (HR: 1.79, 95% CI: 1.36–2.32, *p* < 0.001), grade 3–4 (HR: 2.98, 95% CI: 2.24–3.94, *p* < 0.001), pathologic T-stage > 1, lymph node positivity (HR: 2.46, 95% CI: 1.89–3.18, *p* < 0.001), positive margins (HR: 1.92, 95% CI: 1.55–2.38, *p* < 0.001), and chemotherapy receipt (HR: 1.73, 95% CI: 1.27–2.30, *p* < 0.001) were associated with lower OS on UVA, while female sex (HR: 0.68, 95% CI: 0.55, 0.83, *p* ≤ 0.001) was associated with higher OS.

Given the increased risk of delays from surgery to RT on oncologic outcomes for patients with high-risk HNSCC (positive margins and/or extranodal extension), we assessed the effect of delayed RT initiation in patients with ACC and high-risk features, including high-grade and positive margins. Time from surgery to RT start was not associated with survival on UVA in the 122 patients with tumor grade 3–4 and positive margins (HR: 1.00, 95% CI: 0.93–1.07, *p* = 0.902).

### 3.3. Multivariable Analysis

Multivariable analysis (MVA) for OS is provided in Table 3. On MVA, increased duration of RT (adjusted HR [aHR]: 1.13, 95% CI: 1.03–1.24, *p* = 0.012) remained significantly associated with worse OS on MVA, while TTI (aHR: 1.00, 95% CI: 0.98–1.02, *p* = 0.979) did not.

Additionally, increasing age (aHR: 1.03, 95% CI: 1.02–1.04, *p* < 0.001), sex (aHR: 0.62, 95% CI: 0.50–0.76, *p* < 0.001), urbanity (aHR: 1.43, 95% CI: 1.06–1.94, *p* = 0.021), Medicare insurance (aHR: 1.36, 95% CI: 1.01–1.83, *p* = 0.041), Charlson–Deyo score of 1 (aHR: 1.42, 95% CI: 1.09–1.84, *p* = 0.008), T-stage > 1 (aHRs between 1.62 and 3.00 for T2, 3, and 4, all with *p* ≤ 0.013), node-positivity (aHR: 1.81, 95% CI: 1.36–2.41, *p* < 0.001), positive margins (aHR: 1.54, 95% CI: 1.22–1.95, *p* < 0.001), and receipt of chemotherapy (aHR: 1.40, 95% CI: 1.01–1.94, *p* = 0.042) had significant associations with OS on MVA.

**Figure 2:**
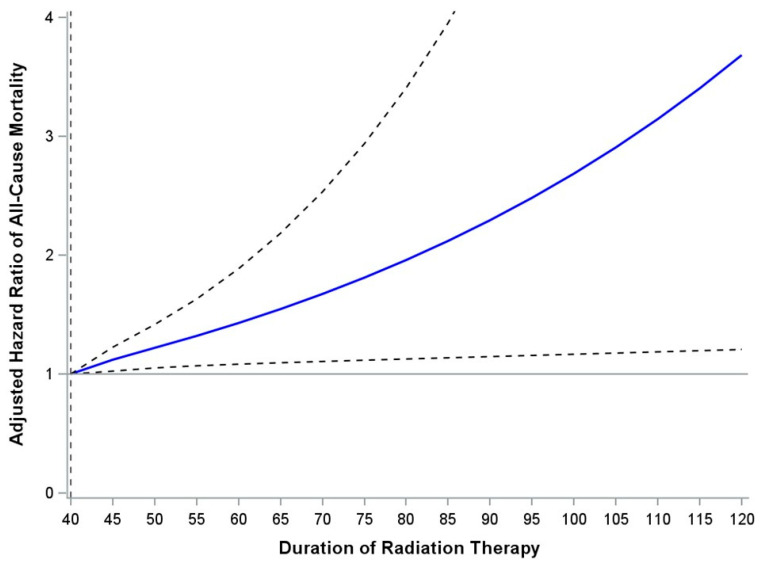
Dose–response graph showing adjusted modeling of all-cause mortality against duration of radiation therapy (RT) (*n* = 1449). Dashed lines represent 95% Confidence Intervals.

After fitting a restricted cubic spline to model the relationship between duration of RT and survival, the results remained consistent. As shown in Supplemental Table S1 and in Figure 2, OS was significantly associated with RT duration at all timepoints beyond 40 days, with HR proportionally increasing with each 5-day increase in RT duration.

Due to the inferior OS seen on UVA and MVA for patients who received chemotherapy, additional analyses were conducted to assess the association of chemotherapy with survival in subgroups. As shown in Supplemental Tables S2 and S3, patients who received chemotherapy had higher pathologic T-stage (adjusted odds ratio [aOR] for pT4: 7.59, 95% CI: 3.68–15.65, *p* < 0.001), N-stage (aOR for pN+: 2.47, 95% CI: 1.61–3.77, *p* < 0.001), and positive margins (aOR: 1.59, 95% CI: 1.09–2.32, *p* = 0.017), in addition to longer duration of radiation (aOR: 1.28, 95% CI: 1.11–1.48, *p* < 0.001). As shown in Supplemental Tables S4–S6, patients who received chemotherapy with pathologic N0 stage (HR: 2.26, 95% CI: 1.49–3.43, *p* < 0.001) or negative margins (HR: 2.25, 95% CI: 1.17–4.32, *p* = 0.015) had significantly worse OS than those who did not receive chemotherapy in those subgroups.

## 4. Discussion

We assessed whether delays in treatment initiation and completion had an impact on outcomes for patients with ACC. We made the following observations: (I) several baseline characteristics were significantly associated with survival on UVA, including age, sex, rurality, insurance, income, Charlson–Deyo score, grade, T- and N-stage, and surgical margins; (II) prolonged time from diagnosis to surgery was associated with worse survival on UVA, with statistical significance; however, the small HR (1.02), in addition to the lack of statistical significance on MVA, suggests that this likely does not carry clinical significance; (III) time from surgery to RT start was not significantly associated with survival, even in patients with high-risk surgical features, including high-grade and positive margins; (IV) increased duration of RT was significantly associated with worse survival on UVA (HR = 1.14) and MVA (HR = 1.13); (V) patients who received chemotherapy had worse survival on UVA and MVA, which was particularly true for those with pN0 stage (HR = 2.26) and/or negative margins (HR = 2.25).

Overall, this analysis suggests that delays from diagnosis to surgery and from surgery to radiation do not have a clinically significant effect on survival. This is in contrast to HNSCC and other malignant salivary gland tumors, in which delays between diagnosis and treatment, from surgery to RT, and from RT initiation to completion can each negatively impact outcomes, as shown in numerous studies [16,17,18,19,20,21,22,23,24,25,26,27,28,29]. In order to combat this risk, efforts have been made to minimize delays through administrative interventions, such as upgrading treatment planning systems and automating certain aspects of clinical workflow [37], and through the use of accelerated fractionation [18,38]. For HNSCC, in which there is sufficient evidence to show the association between delays and outcomes, this is paramount. For ACC, this may not be necessary.

This also calls into question whether the timeframe in which postoperative RT may provide benefit extends beyond that typically quoted for HNSCC. While there is no hard cutoff, most physicians aim to start postoperative RT within 6 weeks of surgery for HNSCC and feel that the benefits of RT begin to diminish when postoperative delays exceed 8–12 weeks. The results from this study suggest this may not be true for ACC, as its slow growth rate may allow for a benefit to be seen even several months after surgery for those who experience delays.

Although TTI and time from surgery to radiation were not associated with survival, we found that a prolonged duration of RT was a significant predictor of worse survival. This finding aligns with previous studies evaluating the impact of treatment timing on outcomes in HNSCC and other salivary gland malignancies [33,39,40,41].

However, the interpretation of this association requires careful consideration of potential confounding and residual bias inherent in retrospective registry analyses. Several mechanisms may underlie an association between prolonged radiation duration and survival. Treatment prolongation can reflect unplanned breaks due to acute toxicity, hospitalization, nutritional decline, or social barriers such as transportation and caregiver limitations. System-level factors including scheduling constraints, staffing shortages, machine downtime, or the need for replanning can also extend treatment duration. In registry data, radiation duration may therefore act as a composite surrogate for both treatment interruptions and broader patient frailty or complication burden, which are not fully captured by comorbidity indices, leaving the possibility of residual confounding.

Several limitations of the NCDB restrict further exploration of the mechanisms underlying this association, such as the benefit of accelerated or hypofractionation. For instance, the therapeutic benefit of RT may be diminished given ACC’s radioresistant nature, particularly in the setting of mid-treatment delays, which may reduce the radiobiologic effectiveness of fractionated RT [42]. Alternatively, prolonged RT duration may serve as a surrogate for factors such as comorbidities, social determinants of health, or issues with patient compliance. Similarly, prolonged RT duration may also reflect treatment-related toxicity, with interruptions or hospitalizations contributing to delays in RT completion.

There remains a dearth of prospective randomized evidence studying the outcomes of chemotherapy for ACC. While some retrospective studies have suggested chemoradiotherapy may be a viable treatment option for locally advanced ACC [43,44,45,46], others have shown low response rates to chemotherapy, suggesting a lack of significant benefit [47,48]. We found that patients who received chemotherapy had lower overall survival on UVA and MVA. While baseline characteristics were less favorable for patients who received chemotherapy, the diminished survival was particularly evident in patients with lower risk surgical features, such as pN0 stage and negative margins. While this study did not show benefit to the addition of chemotherapy, caution should be taken when interpreting these results given its retrospective nature, as it does not represent a meta-analysis of high-quality prospective interventional trials.

This study is subject to the inherent limitations of retrospective analyses. Notably, the NCDB does not capture oncologic-specific outcomes, such as cancer-specific survival and recurrence, limiting our ability to assess whether treatment delays directly impact local control. Our findings regarding the lack of association between early treatment delays (diagnosis to surgery, surgery to RT) and overall survival should be considered hypothesis-generating rather than practice-changing. Although we adjusted for known prognostic variables including tumor stage and comorbidity index, the NCDB lacks other important clinical data, such as performance status and the extent of perineural or soft tissue invasion, which may also influence survival outcomes. Additionally, the database does not provide information regarding the reasons for treatment delays. While our data demonstrate an association between prolonged RT duration and worse survival, the inability to distinguish treatment-related toxicity, patient compliance issues, and biological tumor factors from true causal effects necessitates cautious interpretation. The most defensible clinical implication is that efforts to minimize unplanned RT interruptions remain prudent, though whether this reflects a direct treatment effect or serves as a marker for adverse patient-level factors requires further investigation.

To address these limitations, we have initiated an analysis of patients with ACC treated at our National Cancer Institute-Designated Comprehensive Cancer Center, incorporating baseline clinical variables and oncologic outcomes, including local and distant progression, to evaluate whether treatment delays are associated with increased recurrence risk. Our study will include detailed chart abstraction to capture fractionation, planned versus unplanned breaks, toxicity, postoperative complications, and the specific reasons for delay, in addition to distinguishing intentional acceleration or altered fractionation from unplanned prolongation. Endpoints will include local control, distant control, disease-specific survival, and recurrence.

## 5. Conclusions

Delays in treatment initiation and in the interval from surgery to radiation did not result in clinically significant differences in survival in this analysis, while prolonged duration of radiation therapy was significantly associated with worse survival. These findings are hypothesis-generating and suggest that treatment delays for ACC may have different effects on oncologic outcomes than those for HNSCC; however, prospective data is paramount to verify these results before strong conclusions can be made.

## Figures and Tables

**Table 1:** Patient demographic, clinical, and treatment characteristics (*n* = 1449).

Patient Characteristic	*n* (%)
Age: Median (Q_1_, Q_3_)	58 (47, 67)
Sex: Female	892 (61.6)
Race	
White	1187 (81.9)
Black	139 (9.6)
Asian	89 (6.1)
Other/Unknown	34 (2.3)
Ethnicity	
Non-Hispanic	1291 (89.1)
Hispanic	107 (7.4)
Unknown	51 (3.5)
Rurality	
Metropolitan	1256 (86.7)
Urban	167 (11.5)
Rural	26 (1.8)
Insurance at Diagnosis	
Private Insurance/Managed Care	845 (58.3)
Medicare	434 (30.0)
Medicaid	102 (7.0)
Other Governmental	25 (1.7)
Not Insured	43 (3.0)
Income	
<$46,277	180 (12.4)
$46,277–$57,856	234 (16.1)
$57,857–$74,062	291 (20.1)
≥$74,063	523 (36.1)
Unknown	221 (15.3)
Charlson–Deyo Score	
0	1211 (83.6)
1	179 (12.4)
2	34 (2.3)
≥3	25 (1.7)
Primary Site	
C07.9 (Parotid Gland)	707 (48.8)
C08.0 (Submandibular Gland)	405 (28.0)
C31.0 (Maxillary Sinus)	138 (9.5)
C30.0 (Nasal Cavity)	105 (7.2)
C08.1 (Sublingual Gland)	39 (2.7)
C08.9 (Major Salivary Gland, Non-Specified)	34 (2.3)
C31.1 (Ethmoidal Sinus)	15 (1.0)
C08.8 (Overlapping Lesion of Major Salivary Glands)	6 (0.4)
Grade	
1 or 2	513 (35.4)
3 or 4	218 (15.0)
Unknown	718 (49.6)
Pathological T-Stage	
pT1	363 (25.1)
pT2	351 (24.2)
pT3	381 (26.3)
pT4	354 (24.4)
Pathological N-Stage	
pN0	900 (62.1)
pN+	197 (13.6)
No Lymph Nodes Examined	352 (24.3)
Margins of Primary Site	
Negative	678 (46.8)
Positive	771 (53.2)
Radiation Dose (Gy): Median (Q_1_, Q_3_)	66 (60, 66)
Chemotherapy	163 (11.2)
Follow-up (Months): Median (Q_1_, Q_3_)	66.7 (38.8, 105.4)

Abbreviations: Q_1_, Q_3_ = 1st and 3rd quartiles.

**Table 2:** Univariable analysis for overall survival.

Variable	Crude HR (95% CI)	*p*-Value
Age		<0.001
1-Year Increase	1.04 (1.03–1.05)	
Sex		<0.001
Male	Ref	
Female	0.68 (0.55–0.83)	
Race		0.747
White	Ref	
Black	0.92 (0.64–1.29)	0.653
Asian	0.76 (0.44–1.24)	0.307
Other/Unknown	0.91 (0.39–1.78)	0.816
Ethnicity		0.710
Non-Hispanic	Ref	
Hispanic	1.08 (0.70–1.59)	
Rurality		0.004
Metropolitan	Ref	
Urban	1.54 (1.15–2.03)	0.003
Rural	1.69 (0.84–3.01)	0.092
Insurance		<0.001
Private	Ref	
Medicaid	1.08 (0.66–1.68)	0.730
Medicare	2.48 (2.00–3.08)	<0.001
Not Insured	0.92 (0.42–1.75)	0.832
Other Governmental	1.80 (0.76–3.55)	0.165
Income		0.204
<$46,227	1.46 (1.06–1.99)	0.016
$46,227–$57,856	1.15 (0.84–1.55)	0.386
$57,587–$74,062	1.10 (0.82–1.45)	0.532
$74,063 or higher	Ref	
Unknown	1.10 (0.78–1.51)	0.586
Charlson–Deyo Score		<0.001
0	Ref	
1	1.79 (1.36–2.32)	<0.001
2	1.26 (0.57–2.37)	0.523
≥3	1.80 (0.71–3.71)	0.156
Grade		<0.001
1 or 2	Ref	
3 or 4	2.98 (2.24–3.94)	<0.001
Unknown	1.22 (0.96–1.56)	0.106
Pathological T-Stage		<0.001
pT1	Ref	
pT2	1.93 (1.34–2.82)	<0.001
pT3	2.91 (2.06–4.18)	<0.001
pT4	4.07 (2.92–5.81)	<0.001
Pathological N-Stage		<0.001
pN0	Ref	
pN+	2.46 (1.89–3.18)	<0.001
No Lymph Nodes Examined	1.03 (0.78–1.34)	0.832
Margins of Primary Site		<0.001
Negative	Ref	
Positive	1.92 (1.55–2.38)	
Radiation Dose		0.495
10-Gy Increase	1.10 (0.84–1.43)	
Chemotherapy		<0.001
No	Ref	
Yes	1.73 (1.27–2.30)	
Time from Dx to Surgery		<0.001
7-day Increase	1.02 (1.01–1.03)	
Time from Surgery to RT Start		0.647
7-day Increase	1.01 (0.97–1.04)	
Duration of RT		0.004
7-day Increase	1.14 (1.04–1.25)	

Abbreviations: HR (95% CI) = hazard ratio (95% confidence interval); Dx = diagnosis; RT = radiation therapy.

**Table 3:** Adjusted analysis for time from diagnosis to surgery and duration of radiation therapy.

Variable	Adjusted HR (95% CI)	*p*-Value
Age		<0.001
1-Year Increase	1.03 (1.02–1.04)	
Sex		<0.001
Male	Ref	
Female	0.62 (0.50–0.76)	
Rurality		0.057
Metropolitan	Ref	
Urban	1.43 (1.06–1.94)	0.021
Rural	1.27 (0.67–2.40)	0.465
Insurance		0.329
Private	Ref	
Medicaid	1.04 (0.64–1.68)	0.889
Medicare	1.36 (1.01–1.83)	0.041
Not Insured	0.96 (0.47–1.96)	0.909
Other Government	1.55 (0.60–3.96)	0.364
Charlson–Deyo Score		0.041
0	Ref	
1	1.42 (1.09–1.84)	0.008
2	0.69 (0.32–1.48)	0.343
≥3	1.02 (0.44–2.38)	0.955
Pathological T-Stage		<0.001
pT1	Ref	
pT2	1.62 (1.11–2.36)	0.013
pT3	2.16 (1.51–3.10)	<0.001
pT4	3.00 (2.07–4.35)	<0.001
Pathological N-Stage		<0.001
pN0	Ref	
pN+	1.81 (1.36–2.41)	<0.001
No Lymph Nodes Examined	0.93 (0.71–1.22)	0.613
Margins of Primary Site		<0.001
Negative	Ref	
Positive	1.54 (1.22–1.95)	
Chemotherapy		0.042
No	Ref	
Yes	1.40 (1.01–1.94)	
Time from Dx to Surgery		0.979
7-day Increase	1.00 (0.98–1.02)	
Duration of RT		0.012
7-day Increase	1.13 (1.03–1.24)	

Abbreviations: HR (95% CI) = hazard ratio (95% confidence interval); Dx = diagnosis; RT = radiation therapy.

## Data Availability

Research data are stored in an institutional repository and will be shared upon request to the corresponding author.

## Supplementary Materials

The following supporting information can be downloaded at https://www.mdpi.com/article/10.3390/cancers18050816/s1, Table S1: Association between Duration of Radiation Therapy and Overall Survival; Table S2: Clinical and Treatment Variables Stratified by Treatment with Chemotherapy; Table S3: Adjusted Analysis Showing the Association between Patients Treated with or without Chemotherapy; Table S4: Subgroup Analyses Showing the Association between Overall Survival and Receipt of Chemotherapy by Pathologic T-stage; Table S5: Subgroup Analyses Showing the Association between Overall Survival and Receipt of Chemotherapy by Pathologic N-stage; Table S6: Subgroup Analyses Showing the Association between Overall Survival and Receipt of Chemotherapy by Margins.
