# Supplementary material for: Impact of Treatment Package Time on Survival in Patients with Head and Neck Adenoid Cystic Carcinoma
Source: Cancers (Basel). 2026 Mar 3;18(5):816. doi: 10.3390/cancers18050816 (PMC12984734; doi:10.3390/cancers18050816)
Supplement: Supplementary file 1 [file cancers-18-00816-s001.zip › cancers-4134156-supplementary.pdf]

Suppemental Table S1. Association between Duration of Radiation Therapy and Overall Survival

**Duration of RT** (days)	**HR (95% CI)**	**P-value**
40	Reference	
45	1.12 (1.02-1.23)	0.013
50	1.22 (1.05-1.42)	0.009
55	1.32 (1.07-1.63)	0.010
60	1.43 (1.08-1.88)	0.012
65	1.55 (1.09-2.18)	0.013
70	1.67 (1.11-2.53)	0.015
75	1.81 (1.12-2.94)	0.016
80	1.96 (1.13-3.41)	0.017
85	2.12 (1.14-3.95)	0.018
90	2.29 (1.15-4.59)	0.019
95	2.48 (1.16-5.33)	0.020
100	2.69 (1.17-6.18)	0.020
105	2.91 (1.18-7.18)	0.021
110	3.14 (1.19-8.34)	0.021
115	3.40 (1.20-9.68)	0.022
120	3.68 (1.21-11.24)	0.022

Abbreviations: RT = radiation therapy; HR (95% CI) = hazard ratio (95% confidence interval)

Supplemental Table S2. Clinical and Treatment Variables Stratified by Treatment with Chemotherapy

Variable	All (N = 1449)	RT Only (N = 1286)	Chemo + RT (N = 163)	P-value*
Pathological T-Stage: N (%)				<0.001
pT1	363 (25.1)	354 (27.5)	9 (5.5)	
pT2	351 (24.2)	328 (25.5)	23 (14.1)	
pT3	381 (26.3)	320 (24.9)	61 (37.4)	
pT4	354 (24.4)	284 (22.1)	70 (42.9)	
Pathological N-Stage: N (%)				<0.001
pN0	900 (62.1)	826 (64.2)	74 (45.4)	
pN+	197 (13.6)	150 (11.7)	47 (28.8)	
No Lymph Nodes Examined	352 (24.3)	310 (24.1)	42 (25.8)	
Margins of Primary Site: N (%)				<0.001
Negative	678 (46.8)	632 (49.1)	46 (28.2)	
Positive	771 (53.2)	654 (50.9)	117 (71.8)	
Duration of RT (days): Median (Q_1, Q_3)	46 (43, 50)	46 (43, 50)	48 (45, 52)	<0.001

Abbreviations: RT = radiation therapy; chemo = chemotherapy; Q_1, Q_3 = 1st and 3rd quartiles

* Categorical variables were analyzed using Chi-Squared tests for association; duration of RT was analyzed using a Wilcoxon rank-sum test.

Supplemental Table S3. Adjusted Analysis Showing the Association between Patients Treated with or without Chemotherapy*

Variable	Adjusted OR (95% CI)	P-value
Pathological T-Stage		<0.001†
pT1	[Reference]	
pT2	2.47 (1.12-5.48)	0.025
pT3	5.99 (2.89-12.41)	<0.001
pT4	7.59 (3.68-15.65)	<0.001
Pathological N-Stage		<0.001†
pN0	[Reference]	
pN+	2.47 (1.61-3.77)	<0.001
No Lymph Nodes Examined	1.50 (0.99-2.81)	0.056
Margins of Primary Site		
Negative	[Reference]	
Positive	1.59 (1.09-2.32)	0.017
Duration of RT		
7-Day Increase	1.28 (1.11-1.48)	<0.001

* Patients who received surgery + radiation only were used as the reference group for the outcome.

† Type III p-value tells whether the variable as whole is statistically significant.

Abbreviations: OR = odds ratio; RT = radiation therapy

Supplemental Table S4. Subgroup Analyses Showing the Association between Overall Survival and Receipt of Chemotherapy by Pathologic T-stage*

T-Stage	HR (95% CI)	P-value
pT1	1.20 (0.14-10.14)	0.868
pT2	1.47 (0.60-3.58)	0.402
pT3	1.24 (0.73-2.09)	0.429
pT4	1.44 (0.95-2.18)	0.083

* Patients who received surgery + radiation only were used as the reference group for the outcome.
Abbreviations: HR (95% CI) = hazard ratio (95% confidence interval)

Supplemental Table S5. Subgroup Analyses Showing the Association between Overall Survival and Receipt of Chemotherapy by Pathologic N-stage*

N-Stage	HR (95% CI)	P-value
pN0	2.26 (1.49-3.43)	<0.001
pN+	1.02 (0.61-1.71)	0.949
No Lymph Nodes Examined	1.06 (0.46-2.43)	0.891

* Patients who received surgery + radiation only were used as the reference group for the outcome.
Abbreviations: HR (95% CI) = hazard ratio (95% confidence interval)

Supplemental Table S6. Subgroup Analyses Showing the Association between Overall Survival and Receipt of Chemotherapy by Margins*

Surgical Margins	HR (95% CI)	P-value
Negative	2.25 (1.17-4.32)	0.015
Positive	1.40 (0.99-1.98)	0.054

* Patients who received surgery + radiation only were used as the reference group for the outcome.
Abbreviations: HR (95% CI) = hazard ratio (95% confidence interval)
